# Mortality Prediction from Patient’s First Day PAAC Radiograph in Internal Medicine Intensive Care Unit Using Artificial Intelligence Methods

**DOI:** 10.3390/diagnostics15243138

**Published:** 2025-12-10

**Authors:** Orhan Gok, Türker Fedai Cavus, Ahmed Cihad Genc, Selcuk Yaylaci, Lacin Tatli Ayhan

**Affiliations:** 1 Department of Electrical and Electronics Engineering, Faculty of Engineering, Sakarya University, Sakarya 54050, Turkey; turker.cavus@sakarya.edu.tr; 2 Ahmed Cihad Genc Private Clinic, Istanbul 34480, Turkey; genccihad@gmail.com; 3 Department of Internal Medicine, Faculty of Medicine, Sakarya University, Sakarya 54050, Turkey; selcukyaylaci@sakarya.edu.tr; 4 Department of Radiology, Faculty of Medicine, Sakarya University, Sakarya 54050, Turkey; drlacintatli@gmail.com

**Keywords:** radiography, thoracic, intensive care units, mortality, artificial intelligence

## Abstract

**Introduction:** This study aims to predict mortality using chest radiographs obtained on the first day of intensive care admission, thereby contributing to better planning of doctors’ treatment strategies and more efficient use of limited resources through early and accurate predictions. **Methods:** We retrospectively analyzed 510 ICU patients. After data augmentation, a total of 3019 chest radiographs were used for model training and validation, while an independent, non-augmented test set of 100 patients (100 images) was reserved for final evaluation. Seventy-four (74) radiomic features were extracted from the images and analyzed using machine learning algorithms. Model performances were evaluated using the area under the ROC curve (AUC), sensitivity, and specificity metrics. **Results:** A total of 3019 data samples were included in the study. Through feature selection methods, the initial 74 features were gradually reduced to 10. The Subspace KNN algorithm demonstrated the highest prediction accuracy, achieving AUC 0.88, sensitivity 0.80, and specificity 0.87. **Conclusions:** Machine learning algorithms such as Subspace KNN and features obtained from PAAC radiographs, such as GLCM Contrast, Kurtosis, Cobb angle, Haralick, Bilateral Infiltrates, Cardiomegaly, Skewness, Unilateral Effusion, Median Intensity, and Intensity Range, are promising tools for mortality prediction in patients hospitalized in the internal medicine intensive care unit. These tools can be integrated into clinical decision support systems to provide benefits in patient management.

## 1. Introduction

Mortality rates are significantly high in intensive care units where critically ill patients are monitored [1,2,3,4,5,6,7]. Accurate early predictions allow the clinical team to allocate resources more effectively, increase close monitoring in high-risk cases, and rapidly readjust treatment strategies when necessary, a need also highlighted in recent computational intelligence–based mortality prediction studies [8]. [1,3,4,5,6,7,9]. Chest radiography, especially the postero-anterior chest radiograph (PAAC), plays a critical role in this context and is widely used in the initial evaluation of many pulmonary diseases [10,11,12,13,14,15,16,17,18]. Chest imaging with X-ray can be applied to almost every patient in emergency conditions and intensive care settings due to its low cost, accessibility, rapid acquisition, and short reporting time [10,11,12,13,14,15,16,17,18]. These images, which can be quickly reviewed by clinicians, have the potential to strengthen clinical decision-making processes when combined with artificial intelligence-supported analyses [10,11,12,13,14,15,16,17,18,19,20,21].

In recent years, numerous approaches based on machine learning and deep learning for disease detection and outcome prediction from chest radiographs have been reported in the literature [10,11,12,13,14,15,16,17,18,19,20,21], including studies comparing the performance of different machine learning models in clinical prediction tasks [22]. Some of these studies extract information solely from images, while others combine clinical data with imaging to propose multimodal models [1,6,9,11,14,15,23,24]. In this study, we predicted mortality by analyzing chest images taken within the first 24 h of admission of patients over 18 years old in the Internal Medicine Intensive Care Unit using artificial intelligence methods. The aim was to provide additional prediction when the risk scores generated by the model were used alongside clinical scoring systems. In summary, analyzing chest radiographs taken on the first day of intensive care with artificial intelligence may provide rapid mortality predictions and support clinical decisions. Accordingly, a machine learning model developed on the basis of effective reporting, strong validation, and explainability principles may serve as a practical and sustainable decision-support tool that complements the shortcomings of traditional scoring systems.

## 2. Materials and Methods

Chest radiograph images were collected from patients hospitalized in the internal medicine intensive care unit. A total of 3019 images were analyzed. A single-center, retrospective study was conducted, and a mortality prediction model was developed using chest radiographs taken within the first 24 h of admission to the intensive care unit. The study included non-COVID patients over 18 years of age who were admitted to the internal medicine intensive care unit between 2022 and 2023.

### 2.1. Data Preprocessing

The study included retrospective images of patients taken with a portable X-ray device. Images with appropriate positioning and without or with minimal motion artifacts were included. Chest radiographs were taken in the supine position at a distance not exceeding 1 m using the SG Healthcare Jumong Mobile device available in the hospital.

The dataset consisted of 510 unique patients. For model development, the dataset was randomly split at the patient level into a training/validation set (n = 410) and an independent test set (n = 100). Each patient was assigned to only one of these subsets (training/validation or test), and this splitting was performed before any image augmentation. A test set of 100 patients (approximately 20% of the total dataset) was chosen as a compromise to preserve a sufficiently large sample for model development while still allowing for an independent, patient-level evaluation of generalization performance.

Data augmentation was applied only to the training/validation images to increase variability and reduce overfitting. From each original chest radiograph in the training/validation set, several augmented images were generated using operations such as rotation, horizontal flipping, cropping, brightness and contrast adjustment, Gaussian noise, shear transformation, color and contrast enhancement, and image simplification. This process increased the size of the training/validation set to a total of 3019 images (original + augmented). The 100 test images were not augmented and were used as-is for the final evaluation of model performance.

### 2.2. Ethics Committee Approval

Written permission for the study was obtained from the Non-Interventional Clinical Research Ethics Committee of Sakarya University Training and Research Hospital, where the study was conducted (decision date: 23 February 2024, decision no: 340184-40).

### 2.3. Feature Extraction

A total of 67 radiomic image features were extracted using Python (scikit-image, SciPy, Mahotas, OpenCV, NumPy, Tkinter) and a custom-developed graphical user interface. In addition, 7 clinical variables were obtained after reviewing all data by clinicians.

Image-based features included qualitative radiologic findings (cardiomegaly [cardiothoracic ratio, CTR > 1], unilateral and bilateral infiltration, unilateral and bilateral pleural effusion, pneumothorax, and calcified aorta) and quantitative radiomic descriptors. Quantitative features comprised the cardiothoracic ratio (CTR), Cobb angle, first-order statistics (mean and median intensity, variance, skewness, kurtosis, intensity range, and entropy), 6 gray-level co-occurrence matrix (GLCM) features, and 52 Haralick texture features.

#### Radiologic Feature Extraction

Cobb angle measurement: The Cobb angle was calculated on PAAC chest radiographs by first identifying the most tilted superior and inferior vertebrae in the coronal plane of the spine. The superior and inferior endplates of these vertebrae were manually marked by the operator using a custom image-processing interface. The software then automatically drew straight lines along the selected endplates, constructed perpendicular lines, and computed the intersection angle, which represents the Cobb angle and quantitatively expresses the degree of spinal curvature. In this study, the measurement workflow was semi-automated and implemented using an OpenCV-based coordinate mapping system to ensure reproducible angle extraction directly from digital chest radiographs. A smaller Cobb angle (<10°) is generally considered to reflect normal spinal alignment, whereas higher values indicate increasing degrees of scoliosis or kyphosis; in our analysis, the Cobb angle was treated as a continuous quantitative feature.

Cardiothoracic Ratio (CTR): The cardiothoracic ratio (CTR) was measured on posteroanterior (PA) chest radiographs by calculating the ratio between the maximal horizontal cardiac diameter and the maximal internal thoracic diameter. The cardiac diameter was defined as the sum of the distances from the midline to the most lateral borders of the cardiac silhouette on both sides, whereas the thoracic diameter was measured as the maximal internal width of the thoracic cage at the level of the diaphragm. A CTR value of approximately 0.50 or lower is generally considered to represent a normal cardiac silhouette, whereas values greater than 0.50 are typically associated with cardiomegaly or other forms of structural cardiac enlargement. In this study, CTR measurement was automated using an OpenCV-based coordinate mapping system, ensuring accurate and reproducible quantitative cardiac size estimation directly from digital chest radiographs. The resulting CTR was used as a continuous radiomic feature in the mortality prediction models.

Radiomic and texture features: From each chest radiograph, a set of radiomic and texture-based features was automatically extracted using a custom Python-based tool. First-order histogram statistics (mean intensity, variance, skewness, kurtosis, median intensity, intensity range, and image entropy) were calculated to characterize the overall pixel intensity distribution and brightness variability within the lung region. In addition, gray-level co-occurrence matrix (GLCM) features, such as contrast, homogeneity, energy, correlation, dissimilarity, and angular second moment (ASM), were computed to capture spatial relationships and texture uniformity. Haralick texture features were then derived from GLCMs generated at multiple orientations (0° and 45° in this study), reflecting direction-dependent texture complexity and structural anisotropy. Together, these radiomic descriptors provide a quantitative description of underlying texture patterns that may correspond to pathological changes and serve as input for the machine learning-based mortality prediction models. A complete list and mathematical definitions of all GLCM and Haralick features are provided in the Supplementary Material.

A complete list of all extracted radiomic and texture features, including first-order statistics, GLCM metrics, and Haralick descriptors, is provided in Supplementary Table S1.

A summarized overview of the radiomic feature categories is provided in the Methods section. The full list of all extracted radiomic, GLCM, and Haralick texture features is presented in Supplementary Table S2.

### 2.4. Software and Statistical Analysis

Features were extracted using the Skimage, Scipy 6, Mahotas, CV2, Numpy, Tkinter libraries in the Python 3.12 programming language (Python Software Foundation, Wilmington, DE, USA). Analyses were conducted on MATLAB Online (basic; The MathWorks Inc., Natick, MA, USA), the online platform provided by MathWorks. Ten different machine learning algorithms were used to predict mortality. Model performances were evaluated using the area under the ROC curve (AUC), sensitivity, specificity, precision, and F1 Score.

To reduce dimensionality and prevent overfitting, feature selection was performed exclusively on the training/validation set. First, starting from the initial 74 radiomic and radiologic predictors, we computed the Pearson correlation matrix and removed redundant features with an absolute correlation coefficient $\left|r\right|\geq 0.90$ reducing the feature set to 36 candidates. Second, these 36 predictors were ranked according to a Shapley value (SHAP) based global importance measure (mean absolute Shapley value), and nested models including the top 15, 10 and 4 predictors were evaluated. The independent test set was not used in any feature selection step and was reserved solely for the final performance assessment of the trained models.

In addition, to tune model hyperparameters and evaluate the stability of model performance, k-fold cross-validation (k = 5) was applied within the training/validation set using the MATLAB Classification Learner application. The independent test set was not used during any stage of feature selection, cross-validation, or model training; it was reserved solely for the final performance evaluation of the trained models.

**Figure 1:**
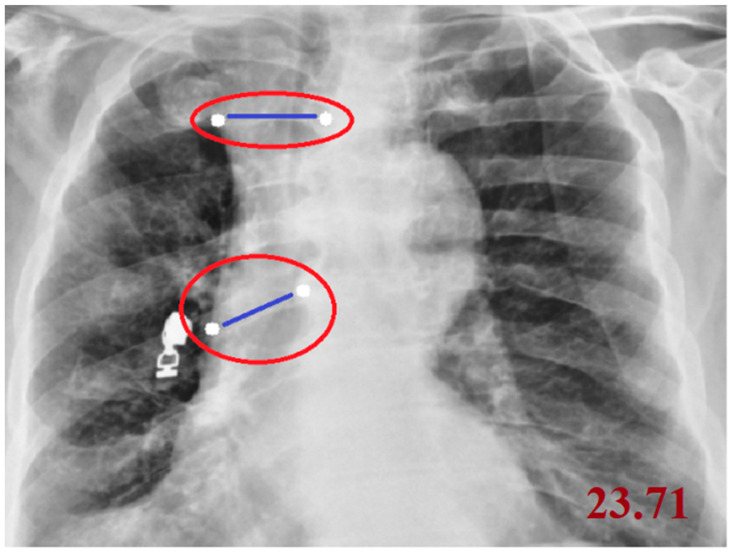
Measurement of the Cobb Angle on a Posteroanterior Chest Radiograph.

Cobb angle measurement on a posteroanterior chest radiograph. The most tilted superior and inferior vertebrae are identified, lines are drawn along their endplates, and the angle between the perpendiculars quantifies the degree of spinal curvature. The white points denote the start and end landmarks used for the measurement, the blue lines represent the distance between these points, and the red ellipses highlight the annotated measurement regions (Figure 1).

**Figure 2:**
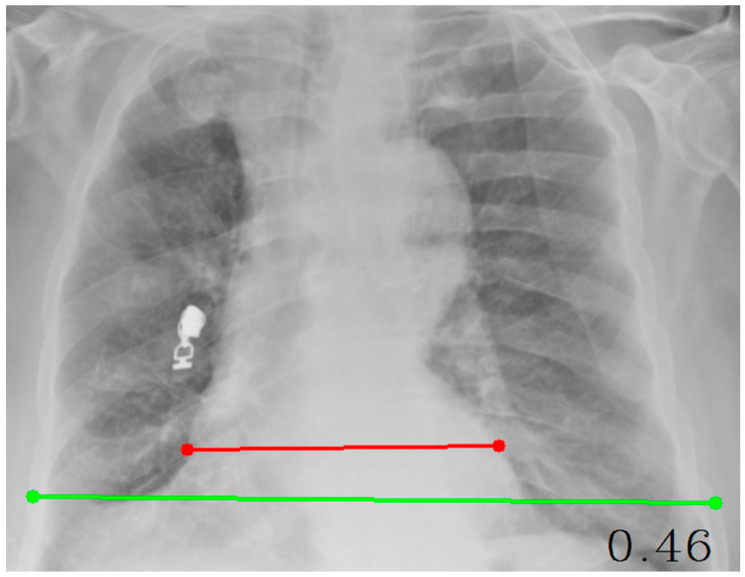
Measurement of the Cardiothoracic Ratio (CTR) on a Posteroanterior Chest Radiograph.

Cardiothoracic ratio (CTR) measurement on a posteroanterior chest radiograph. The maximal horizontal cardiac diameter (red line) is divided by the maximal internal thoracic diameter (green line) to obtain the CTR value (Figure 2).

This table summarizes the Python-based computational modules developed for quantitative image analysis. Each module is designed for a specific radiological measurement or feature extraction process (Table 1).

The CTR (Cardiothoracic Ratio) module calculates the ratio between the cardiac and thoracic widths using user-selected points on posteroanterior chest radiographs. The Cobb Angle module determines vertebral tilt and spinal curvature by measuring the angle formed between two selected vertebral lines. Finally, the Feature Extraction module employs advanced image-processing libraries such as scikit-image, SciPy, and Mahotas to extract texture-based (GLCM and Haralick) and statistical radiomic features, which are essential for machine learning–based diagnostic and prognostic modeling.

After this stage, analyses were conducted using the Classification Learner toolbox in MATLAB R2023b with a total of 74 extracted features. Among the 510 patient datasets, 410 were used for training and 100 for testing. Images belonging to the training group were augmented using various methods such as rotation, flipping, cropping, brightness adjustment, contrast adjustment, Gaussian noise addition, shearing, color conversion, contrast enhancement, and simplification. From each original X-ray image, 4–6 augmented images were generated, resulting in a total of 3019 training images and 100 test images used in the analyses. The analyses were evaluated using multiple performance assessment methods, including the ROC Curve, Confusion Matrix, Evaluation Metrics (AUC, F1-Score, Sensitivity, Specificity), Shapley-based Feature Selection, and Shapley Summary Analysis.

### 2.5. Classification and Model Training

All classification models were developed using the Classification Learner app in MATLAB R2023b (MathWorks, Natick, MA, USA). Model development and parameter tuning were performed exclusively on the training/validation set, with no information from the independent test set used during this process. Within the app, we evaluated a broad set of supervised learning algorithms, including fine/medium/coarse k-nearest neighbors (KNN), cosine and cubic KNN, linear/quadratic/cubic support vector machines (SVM), decision trees, bagged trees, boosted trees, and RUSBoost ensemble methods.

For all models, 5-fold cross-validation was applied on the training/validation data to estimate generalization performance and guide hyperparameter selection. Models were primarily compared and selected based on cross-validated AUC values. Among the tested algorithms, the best performance was obtained with a Subspace KNN ensemble classifier. This model used a subspace dimension of 5 predictors, a total of 30 learners, and KNN as the base learner in each subspace. The distance metric was left at the MATLAB default (Euclidean), and all predictor variables were standardized before training. After cross-validation, the final Subspace KNN model was exported from the Classification Learner app to the MATLAB workspace and evaluated only once on the independent, non-augmented test set.

## 3. Results

### 3.1. Analyses

#### 3.1.1. Performance with 74 Features

First, analyses were performed using the Classification Learner tool in MATLAB software with 3019 training data, 100 test data, and 74 features. In the analyses, the best features were selected, and machine learning algorithms with results of 0.80 and above were considered successful. As a result of the analysis, the Weighted KNN algorithm achieved values of AUC 0.91, F1 Score 0.85, Sensitivity 0.79, and Specificity 0.90. The SVM Kernel algorithm yielded AUC 0.87, F1 Score 0.83, Sensitivity 0.79, and Specificity 0.82 values; the Medium KNN algorithm yielded AUC 0.88, F1 Score 0.83, Sensitivity 0.80, and Specificity 0.80 values; the Quadratic SVM algorithm yielded AUC 0.87, F1 Score 0.80, Sensitivity 0.71, and Specificity 0.90 values; and the Cosine KNN algorithm yielded AUC 0.87, F1 Score 0.83, Sensitivity 0.82, and Specificity 0.74 values. These algorithms were found to be the five algorithms that demonstrated the best performance.

#### 3.1.2. Feature Selection and Performance with 15 Selected Features

From the initial set of 74 radiomic and radiologic features, we first applied a correlation-based pre-filtering step on the training/validation data. We computed the Pearson correlation matrix and defined pairs of predictors with an absolute correlation coefficient $\left|r\right|\geq 0.90$ as highly correlated. Within each such pair, only one predictor was retained (the one showing better univariable discrimination between survivors and non-survivors), while the other was discarded. This procedure reduced the feature set from 74 to 36 candidate predictors.

In the second step, these 36 predictors were ranked using a Shapley value (SHAP) based global importance measure (mean absolute Shapley value) obtained from the machine learning models. The 15-feature model corresponded to the top 15 predictors in this SHAP-based ranking, namely: GLCM Contrast, Kurtosis, Cardiomegaly, Cobb angle, Median Intensity, Haralick_5_90, Bilateral Infiltrates, Skewness, Intensity Range, Unilateral Effusion, Bilateral Effusions, Unilateral Infiltrate, Aortic Calcification, Pneumothorax, and CTR. Feature selection was performed exclusively on the training/validation set to reduce dimensionality, minimize the risk of overfitting, and avoid data leakage from the independent test set.

In subsequent analyses, these 15 SHAP-ranked predictors were used to build nested models including the top 4, 10, and 15 features, and their cross-validated performance was compared. The 4-feature model represented a very compact but less discriminative configuration, whereas the 10-feature model provided a better balance between simplicity and predictive performance, and was therefore selected as the final feature set. The independent test set was not used at any stage of feature selection and was reserved solely for the final evaluation of the trained models.

For Medium Gaussian SVM, the values of AUC 0.81, F1 Score 0.70, Sensitivity 0.62, and Specificity 0.75 were obtained, identifying it as the most successful algorithm at this 15-feature stage.

**Figure 3:**
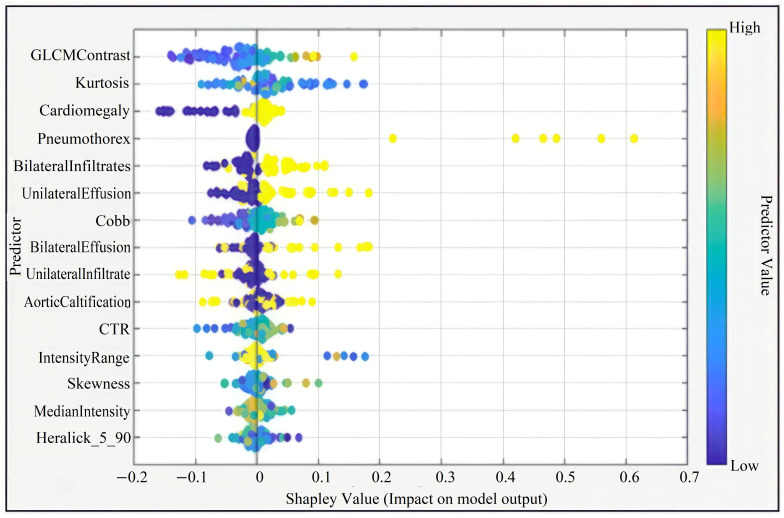
Shapley Summary (for 15 Features).

This figure presents the Shapley summary plot illustrating the relative importance and contribution of each predictor variable to the model output. Predictors such as GLCM Contrast, Kurtosis, Cardiomegaly, and Pneumothorax had the highest impact on model predictions. The color gradient from blue to yellow indicates the variation in feature values from low to high (Figure 3).

#### 3.1.3. Performance with 10 Optimal Features

Five features deemed ineffective were removed, and the analysis was continued with 10 features (GLCM Contrast, Kurtosis, Cobb angle, Haralick 5_90, Bilateral Infiltrates, Cardiomegaly, Skewness, Intensity Range, Unilateral Effusion, Median Intensity). For Subspace KNN, the results were AUC 0.88, F1 Score 0.82, Sensitivity 0.80, and Specificity 0.87, making it the best-performing algorithm.

Among all algorithms tested with the 10 selected features in the MATLAB Classification Learner application, the Subspace KNN ensemble classifier achieved the best performance. This model demonstrated stable generalization across folds by achieving high accuracy both during cross-validation and on the independent test set. The classifier used a subspace dimension of 5, a total of 30 learners, and the k-nearest neighbors (KNN) method within each learner. The distance metric remained set to MATLAB’s default Euclidean value, and all predictor variables were standardized.

This model outperformed all other evaluated algorithms, including the fine/medium/coarse KNN variants, cosine and cubic KNN, linear/quadratic/cubic SVMs, decision trees, and ensemble methods such as bagged trees, boosted trees, and RUSBoost. After being identified as the best-performing classifier, the model was exported to the MATLAB workspace and evaluated exclusively on the independent test set.

**Figure 4:**
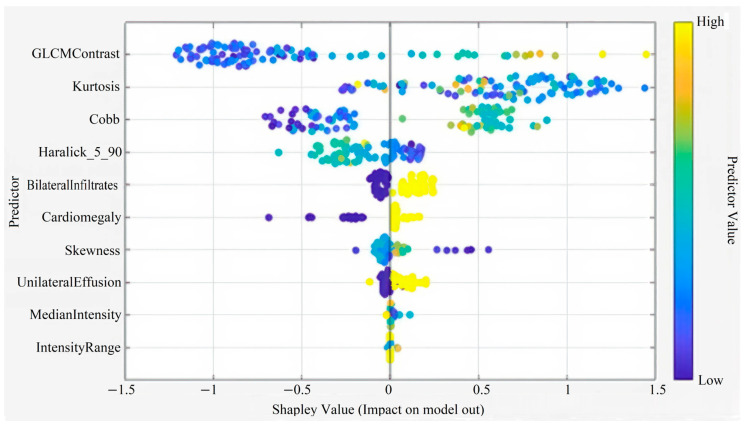
Shapley Summary (for 10 Features).

This figure illustrates the Shapley summary plot showing the relative contribution and impact of each predictor variable on the model’s output. Features such as GLCM Contrast, Kurtosis, Cobb angle, and Haralick_5_90 demonstrated the strongest influence on model predictions. The color gradient from blue to yellow represents the predictor value range from low to high (Figure 4).

#### 3.1.4. Performance with 4 Core Features

Further analysis showed that six more features were ineffective, and the analysis was repeated with the best four features (GLCM Contrast, Cobb angle, Haralick 5_90, Kurtosis). For the Boosted Trees algorithm, the results were AUC 0.80, F1 Score 0.72, Sensitivity 0.67, and Specificity 0.80, making it the best-performing algorithm at this stage.

**Figure 5:**
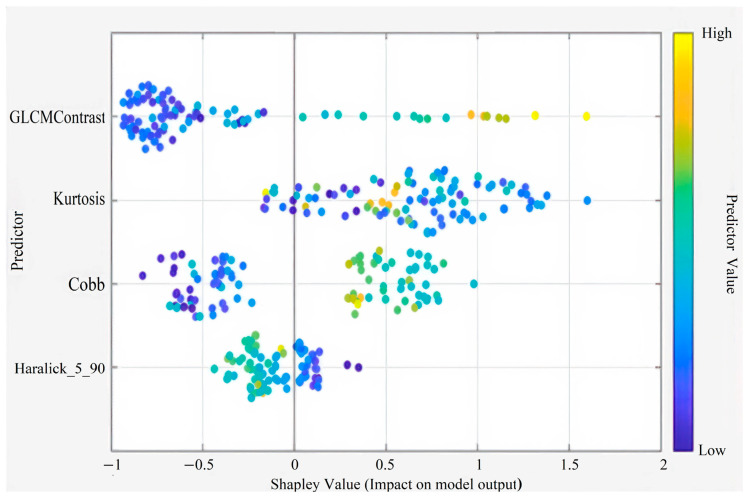
Shapley Summary Plot (for 4 Features).

This figure illustrates the Shapley summary plot highlighting the four most influential predictors in the model. Among these, GLCM Contrast, Kurtosis, Cobb angle, and Haralick_5_90 showed the greatest contribution to model predictions. The color gradient from blue to yellow represents the predictor value range from low to high, indicating how each variable influences the model’s output direction and magnitude (Figure 5).

#### 3.1.5. Overall Performance Summary

The aim of the analyses was to achieve the highest performance with the least number of features. With 10 features, an AUC of 0.88 was reached, and with 4 features, an AUC of 0.80 was obtained. In conclusion, when all performance metrics were evaluated together, it was determined that the Subspace KNN algorithm produced the best results with an analysis conducted using 3019 training data, 100 test data, and the best 10 features, achieving AUC 0.88, Sensitivity 0.80, Specificity 0.87, and F1 Score 0.82.

Table 2 provides a summary of the best-performing classifier’s performance on the independent test set using different feature subsets (74, 15, 10, and 4 features). AUC, sensitivity, and specificity are reported for each configuration.

**Figure 6:**
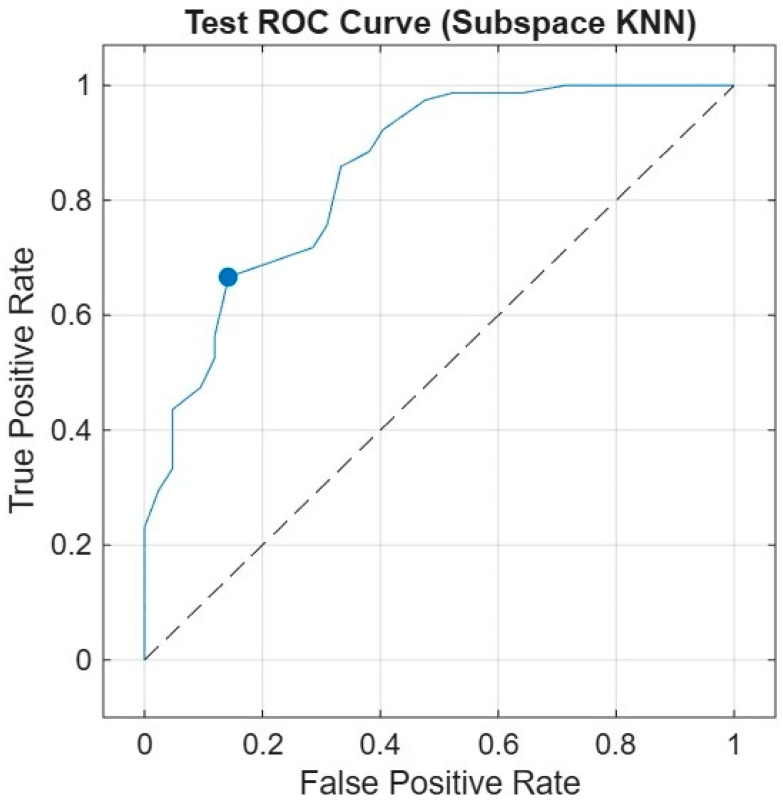
Receiver Operating Characteristic (ROC) Curve.

Test ROC curve of the Subspace KNN classifier using the 10-feature model on the independent test set. The curve shows the relationship between the true positive rate (sensitivity) and the false positive rate (1—specificity). The model achieved an AUC of 0.88, indicating high discriminatory power in distinguishing between survivors and non-survivors (Figure 6).

## 4. Discussion

The present study demonstrates that radiomic and radiologic features extracted from first-day PAAC chest radiographs can be used to predict ICU mortality with good discriminative performance in an internal medicine ICU population. Among the 74 features obtained from chest radiographs, the 10 most effective features (GLCM Contrast, Kurtosis, Cobb angle, Haralick, Bilateral Infiltrates, Cardiomegaly, Skewness, Intensity Range, Unilateral Effusion, Median Intensity) were identified. By comparing the results of 10 different machine learning algorithms, the highest success metrics were achieved with Subspace KNN. This model achieved an AUC of 0.88 on an independent, non-augmented test set of 100 patients, suggesting that quantitative CXR-derived biomarkers may support early risk stratification at the time of ICU admission.

In addition to the feature selection procedure, we performed a focused analysis to explain why the most influential predictors such as GLCM contrast, kurtosis, Cobb angle, and cardiomegaly may be associated with mortality risk. GLCM contrast captures heterogeneity and edge prominence within the lung fields, which tends to increase in severe infiltrative, edematous, or fibrotic processes. Kurtosis reflects the peakedness of the intensity distribution and becomes abnormal in lungs with consolidation, hyperinflation, or mixed radiographic patterns. The Cobb angle provides a proxy for thoracic deformity, which can mechanically impair ventilation and reduce cardiopulmonary reserve. Cardiomegaly, quantified through the cardiothoracic ratio (CTR), represents increased cardiac workload and structural cardiac alteration and has been widely associated with adverse outcomes in critically ill patients. Taken together, these imaging-derived markers likely reflect the combined cardiopulmonary burden and structural lung damage, which may explain their high importance in our ICU mortality prediction model.

We also examined misclassified cases (false positives and false negatives) to better understand the limitations of our model. Many of these errors occurred in radiographs with borderline or overlapping patterns, such as mild, patchy infiltrates combined with minimal cardiomegaly or non-specific interstitial changes. In such cases, patients received predicted risk scores close to the decision threshold, and visually similar images could be assigned to either the survivor or non-survivor group. This observation suggests that, for complex or equivocal presentations, relying solely on image-based features may be insufficient and that future multimodal models integrating clinical and laboratory variables will be needed to further improve discrimination and robustness.

In addition, one of the main motivations of our work was to explore whether accurate mortality prediction could be achieved using the simplest and most widely available imaging modality in the ICU: the routine chest radiograph. Chest X-rays are inexpensive, rapidly obtainable at the bedside, and almost universally accessible, whereas laboratory panels and more advanced imaging modalities may have variable turnaround times and are not always immediately available at admission. By focusing primarily on CXR-derived features, we aimed to demonstrate that an interpretable and relatively simple model, built on a single standard radiograph, can still provide meaningful prognostic information without requiring complex multimodal inputs. This simplicity may be particularly advantageous in resource-limited settings and can serve as a practical complementary tool to be integrated with clinical and laboratory data in future multimodal frameworks.

Traditionally, ICU mortality prediction has relied on clinical scoring systems such as APACHE II, SAPS II and comorbidity-based indices. Several studies have shown that these scores, while widely adopted, present variable performance across patient groups and require multiple physiological and laboratory measurements that may not always be immediately available [1,2,3,4,5,6,7]. In parallel, there has been a growing interest in machine-learning-based models that use electronic medical records and clinical data to improve outcome prediction in both COVID-19 and non–COVID settings [1,9,25]. For example, Estiri et al. [9] used EMR data to predict COVID-19 mortality with good discrimination, and Bardak and Tan [25] developed deep learning models to predict mortality and length of stay. Genc et al. [1] specifically focused on an internal medicine ICU cohort and showed that machine learning models based on clinical variables can provide accurate mortality prediction. More recently, Tu et al. [6] reported that a LightGBM-based model for traumatic brain injury patients admitted to the ICU outperformed traditional scores such as APACHE II and SOFA for early mortality prediction, while Rammos et al. [7] demonstrated that established prognostic scores show heterogeneous accuracy in cardiac ICU populations, underscoring the need for complementary or improved tools. In this context, our model differs from most prior approaches by relying primarily on radiographic texture and morphological features from first-day PAAC images, with only minimal clinical input, and still achieving an AUC of 0.88.

Multiple recent studies have highlighted that chest radiographs contain prognostic information beyond standard visual interpretation. Weiss et al. [12] developed a deep learning model to estimate lung disease–related mortality from chest radiographs and showed that higher risk scores derived from CXRs were strongly associated with adverse outcomes. In community-acquired pneumonia, Kim et al. [13] used a CXR-based deep learning model to predict 30-day mortality with an AUC of 0.80, which further improved when combined with the CURB-65 score. During the COVID-19 pandemic, several groups demonstrated that image-based models derived from chest X-rays can predict mortality or disease severity. Iori et al. [11] developed a radiomic and neural-network-based model using chest X-ray features to predict COVID-19 mortality, achieving an AUC around 0.79. Baik et al. [14] combined initial COVID-19 chest radiographs with electronic health record data to build an ensemble model with AUROC values around 0.87. Olowolayemo et al. [10] showed that chest X-ray images alone can be used to predict COVID-19 mortality in a relatively small dataset. Lin et al. [15] further demonstrated that multimodal models integrating clinical parameters with chest X-rays can enhance 30-day mortality prediction in ICU patients. In contrast to these largely deep-learning–based and frequently COVID-specific approaches, our study extends the concept of CXR-based risk prediction to a non–COVID, mixed internal medicine ICU cohort, using handcrafted radiomic features and conventional machine learning rather than deep neural networks, and still achieving competitive performance.

Clinical and laboratory biomarkers also play an important role in ICU prognostication. Ince et al. [26] showed that age, procalcitonin, neutrophil-to-lymphocyte ratio, platelet-to-lymphocyte ratio and ferritin–lactate indices are significant mortality predictors in COVID-19 ICU patients. Other machine-learning models based on clinical, laboratory or severity scores have demonstrated that routinely available variables can be used to build accurate mortality prediction models in mechanically ventilated patients, critically ill cancer patients, or after non-cardiac surgery [3,4,5,27,28]. In our study, although multiple clinical variables were initially considered, only one remained in the final model after feature selection. Nevertheless, combining this limited clinical information with a rich set of radiomic features extracted from chest radiographs yielded an AUC comparable to many purely clinical or multimodal models, suggesting that imaging-derived biomarkers can complement rather than replace traditional clinical predictors.

Beyond mortality alone, artificial intelligence is increasingly being integrated into ICU workflows for monitoring, early warning, dynamic risk estimation and decision support. Kołodziejczak et al. [29] reviewed the present and future applications of AI in the ICU, particularly in the COVID-19 era, and emphasized its potential to enhance patient monitoring, prognostic modeling and resource allocation. Similar trends are visible in other domains, including pediatric pneumonia, lung cancer detection, opportunistic sarcopenia screening and pneumonia identification from chest X-rays, where CNNs, transformers and other deep learning architectures have achieved high diagnostic performance [13,16,17,18,20,30,31]. Our work aligns with these developments by proposing a fully automated, image-based risk stratification tool that could be implemented using routine ICU chest radiographs without the need for additional imaging protocols or complex infrastructure.

Radiomic approaches have been successfully applied to a wide range of medical imaging tasks and modalities. GLCM- and Haralick-based texture features, in particular, provide a quantitative description of image heterogeneity and have been shown to correlate with clinically relevant outcomes in various disease domains [32,33,34,35]. Texture and radiomics methods have been used in mammography, CT, MRI and other imaging-based prognostic or classification tasks, such as breast cancer detection, lung nodule characterization, rectal cancer staging and musculoskeletal or degenerative diseases [17,31,33,34,36]. Our findings are in line with this literature: several GLCM and Haralick-derived measures (e.g., contrast and direction-specific texture descriptors) emerged among the most important predictors of ICU mortality in our model, indicating that handcrafted texture features remain relevant and informative despite the rise in deep learning.

From a methodological perspective, many studies have compared different machine learning algorithms across diverse medical prediction tasks, including pulmonary disease, thyroid disease, osteoporosis, diabetes, cardiovascular disease and Alzheimer’s disease [20,24,27,28,30,37,38]. These works consistently show that careful feature engineering, feature selection and algorithm comparison are crucial for achieving robust performance. Our results support this view: performances obtained with all 74 features and those achieved after stepwise feature selection differed notably, and a relatively simple classifier—Subspace KNN—outperformed several more complex models once the most informative radiomic features were retained and redundant or noisy variables were removed. In addition, general reviews of feature extraction techniques highlight the importance of choosing appropriate descriptors and dimensionality-reduction strategies for pattern analysis in medical images [39].

Issues related to class imbalance and evaluation methodology are also critical in medical datasets. Mundra et al. [35] provided an empirical analysis of different approaches for handling imbalanced medical data and showed that resampling strategies and appropriate performance metrics are essential for fair model assessment. Although we did not apply advanced cost-sensitive learning or resampling schemes in this study, we attempted to mitigate overfitting by performing feature selection only on the training/validation set, applying k-fold cross-validation within the training data, and reserving an independent, non-augmented test set for final evaluation. Future work could incorporate more sophisticated imbalance-handling methods and hybrid radiomics–deep-learning models to further improve generalization.

Overall, this study indicates that quantitative texture and morphological features derived from routine first-day PAAC chest radiographs can provide meaningful prognostic information for internal medicine ICU patients. Such an approach offers a rapid, inexpensive and non-invasive complement to existing clinical scoring systems and EMR-based prediction models. Rather than replacing established tools, CXR-based risk stratification may serve as a practical component of multimodal decision-support frameworks, helping to identify high-risk patients early, prioritize monitoring and interventions, and support communication about prognosis. Larger multicenter studies and external validation, together with the integration of additional clinical and laboratory variables and longitudinal imaging, will be essential to confirm these findings and further improve the accuracy and generalizability of imaging-based mortality prediction models.

### Limitations

This study has several limitations. First, it was conducted in a single-center ICU population, which may restrict the generalizability of the findings to other hospitals and patient profiles. Second, the independent test set included only 100 patients (approximately 20% of the dataset), limiting the statistical precision of the reported performance metrics. Third, although data augmentation improved model robustness, augmented images are not fully independent samples and cannot replace external validation. Fourth, only one clinical variable remained in the final model after feature screening, which may limit the clinical depth of the prediction and should be expanded in future datasets. Fifth, all radiomic measurements, CTR, Cobb angle, and texture features, were computed from first-day PAAC radiographs only; incorporating CT imaging, temporal progression, or multimodal data could further improve prediction accuracy. Finally, external, multicenter validation studies with larger sample sizes are required to confirm the generalizability of our imaging-based mortality prediction approach.

## 5. Conclusions

As a result of the study, it was observed that mortality prediction of patients hospitalized in intensive care units could be achieved from postero-anterior chest radiographs using machine learning algorithms. The study showed that different features extracted from the images significantly affected performance, and that texture-based features such as GLCM and Haralick-derived metrics, which have not been extensively explored in similar ICU mortality studies, made a notable contribution to model performance.

In particular, the Subspace KNN algorithm provided higher accuracy compared to classical scoring systems in predicting mortality from the first-day PAAC radiograph of patients in the Internal Medicine Intensive Care Unit. Machine learning based models are powerful tools for early mortality prediction and can be integrated into clinical decision support systems. This integration can improve patient risk stratification and optimize resource utilization.

In future work, these findings should be validated in larger, multicenter ICU cohorts with more heterogeneous case mixes. Extending the models to incorporate longitudinal imaging beyond the first ICU day, together with additional clinical and laboratory variables, may further improve predictive performance and clinical usefulness. Moreover, integrating deep learning–based image representations with handcrafted radiomic features and prospectively evaluating the impact of image-based mortality prediction on clinical decision-making and patient outcomes represent important directions for future research.

## Figures and Tables

**Table 1:** Summary of Python Code Modules, Libraries, and Functional Roles.

Code Type	Libraries Used	Purpose/Task	Additional Notes
CTR (Cardiothoracic Ratio)	cv2, numpy, tkinter	Calculates the ratio of cardiac width to thoracic width	Involves point selection and distance measurement
Cobb Angle	cv2, numpy, math, tkinter	Measures vertebral tilt and spinal curvature angle	Calculated using four manually selected points
Feature Extraction (GLCM/Haralick)	skimage, scipy, mahotas, numpy, cv2	Extracts image texture and statistical features	Used for radiomic and texture-based analysis

**Table 2:** Performance metrics.

Performance Metrics							
Number of Features	Best-Performing Algorithms	AUC	Sensitivity	Specificity	Precision	Recall	F1 Score
74	WEİGHTED KNN	0.91	0.79	0.90	0.92	0.79	0.85
74	SVM KERNEL	0.87	0.79	0.82	0.87	0.79	0.83
74	MEDİUM KNN	0.88	0.80	0.80	0.86	0.80	0.83
74	QUADRATİC SVM	0.87	0.71	0.90	0.92	0.71	0.80
74	COSİNE KNN	0.87	0.82	0.74	0.83	0.74	0.83
15	M. GAUSSİAN SVM	0.81	0.62	0.74	0.79	0.62	0.70
15	SUBSPACE KNN	0.79	0.62	0.77	0.81	0.62	0.70
15	BOOSTED TREES	0.79	0.75	0.74	0.82	0.75	0.74
15	BAGGED TREES	0.77	0.71	0.69	0.77	0.77	0.94
15	QUADRATİC SVM	0.79	0.62	0.82	0.84	0.62	0.72
10	SUBSPACE KNN	0.88	0.80	0.87	0.82	0.61	0.82
10	BAGGED TREES	0.75	0.69	0.69	0.80	0.71	0.75
10	BOOSTED TREES	0.76	0.67	0.80	0.81	0.79	0.80
4	BAGGED TREES	0.77	0.69	0.69	0.81	0.71	0.75
4	RUSBoosted TREES	0.76	0.66	0.85	0.87	0.66	0.75
4	BOOSTED TREES	0.80	0.67	0.80	0.84	0.67	0.75

## Data Availability

The data used in this study were obtained from the Sakarya University Training and Research Hospital database. Due to ethical and privacy restrictions, these data are not publicly available but can be provided upon reasonable request from the corresponding author, in compliance with institutional policies.

## Supplementary Materials

The following supporting information can be downloaded at: https://www.mdpi.com/article/10.3390/diagnostics15243138/s1. Supplementary Table S1: Full list of extracted radiomic and texture features used in this study, including first-order statistics, GLCM features, and Haralick descriptors. Supplementary Table S2: Complete list of all radiomic, GLCM, and Haralick features used in the study.

## References

[1] Genc A.C., Özmen E., Cekic D., Issever K., Türkoğlu Genc F., Genc A.B., Toçoğlu A., Durmaz Y., Özkök H., Yaylacı S. (2025). Comprehensive Analyses Using Machine Learning Models for Mortality Prediction in the Intensive Care Unit of Internal Medicine. J. Investig. Med..

[2] Bulur O., Kaplan Efe F., Ispir Iynem H.K., Koc S., Beyan E. (2022). Comparison of APACHE II and Modified Charlson Index in Mortality Prediction in Patients at Medical Intensive Care Unit. Osman. J. Med..

[3] Ko R.-E., Cho J., Shin M.-K., Oh S.W., Seong Y., Jeon J., Jeon K., Paik S., Lim J.S., Shin S.J. (2023). Machine Learning-Based Mortality Prediction Model for Critically Ill Cancer Patients Admitted to the Intensive Care Unit (CanICU). Cancers.

[4] Kim J.H., Kwon Y.S., Baek M.S. (2021). Machine Learning Models to Predict 30-Day Mortality in Mechanically Ventilated Patients. J. Clin. Med..

[5] Choi B., Oh A.R., Lee S.-H., Lee D.Y., Lee J.-H., Yang K., Kim H.Y., Park R.W., Park J. (2022). Prediction Model for 30-Day Mortality after Non-Cardiac Surgery Using Machine-Learning Techniques Based on Preoperative Evaluation of Electronic Medical Records. J. Clin. Med..

[6] Tu K.-C., Tau E., Chen N.-C., Chang M.-C., Yu T.-C., Wang C.-C., Liu C.-F., Kuo C.-L. (2023). Machine Learning Algorithm Predicts Mortality Risk in Intensive Care Unit for Patients with Traumatic Brain Injury. Diagnostics.

[7] Rammos A., Bechlioulis A., Chatzipanteliadou S., Sioros S.A., Floros C.D., Stamou I., Lakkas L., Kalogeras P., Bouratzis V., Katsouras C.S. (2024). The Role of Prognostic Scores in Assessing the Prognosis of Patients Admitted in the Cardiac Intensive Care Unit: Emphasis on Heart Failure Patients. J. Clin. Med..

[8] Khan I.U., Aslam N., Aljabri M., Aljameel S.S., Kamaleldin M.M.A., Alshamrani F.M., Chrouf S.M.B. (2021). Computational Intelligence-Based Model for Mortality Rate Prediction in COVID-19 Patients. Int. J. Environ. Res. Public Health.

[9] Estiri H., Strasser Z.H., Klann J.G., Naseri P., Wagholikar K.B., Murphy S.N. (2021). Predicting COVID-19 Mortality with Electronic Medical Records. NPJ Digit. Med..

[10] Olowolayemo A., Shams W.K., Omer A.Y.I., Mohammed Y., Batha R.S. (2023). COVID-19 Mortality Risk Prediction Using Small Dataset of Chest X-ray Images. Artif. Intell. Appl..

[11] Iori M., Di Castelnuovo C., Verzellesi L., Meglioli G., Lippolis D.G., Nitrosi A., Monelli F., Besutti G., Trojani V., Bertolini M. (2022). Mortality Prediction of COVID-19 Patients Using Radiomic and Neural Network Features Extracted from a Wide Chest X-ray Sample Size. Appl. Sci..

[12] Weiss J., Raghu V.K., Bontempi D., Christiani D.C., Mak R.H., Lu M.T., Aerts H.J. (2023). Deep Learning to Estimate Lung Disease Mortality from Chest Radiographs. Nat. Commun..

[13] Kim C., Hwang E.J., Choi Y.R., Choi H., Goo J.M., Kim Y., Choi J., Park C.M. (2023). A Deep Learning Model Using Chest Radiographs for Prediction of 30-Day Mortality in Patients with Community-Acquired Pneumonia. Am. J. Roentgenol..

[14] Baik S.M., Hong K.S., Park D.J. (2023). Deep Learning Approach for Early Prediction of COVID-19 Mortality Using Chest X-ray and Electronic Health Records. BMC Bioinform..

[15] Lin J., Yang J., Yin M., Tang Y., Chen L., Xu C., Zhu S., Gao J., Liu L., Liu X. (2024). Development and Validation of Multimodal Models to Predict the 30-Day Mortality of ICU Patients Based on Clinical Parameters and Chest X-rays. J. Digit. Imaging.

[16] Ravi V., Narasimhan H., Pham T.D. (2022). A Cost-Sensitive Deep Learning-Based Meta-Classifier for Pediatric Pneumonia Classification Using Chest X-Rays. Expert Syst..

[17] Ma Y., Lv W. (2022). Identification of Pneumonia in Chest X-Ray Image Based on Transformer. Int. J. Antennas Propag..

[18] Ryu J., Eom S., Kim H.C., Kim C.O., Rhee Y., You S.C., Hong N. (2023). Chest X-ray-based opportunistic screening of sarcopenia using deep learning. J. Cachexia Sarcopenia Muscle.

[19] Sitaula C., Shahi T.B., Marzbanrad F., Aryal S. (2021). Automated Deep Learning Framework for COVID-19 Detection Using Chest X-Ray Images: A Novel Approach. Int. J. Environ. Res. Public Health.

[20] Ibrahim D.M., Elshennawy N.M., Sarhan A.M. (2021). Deep-chest: Multi-classification deep learning model for diagnosing COVID-19, pneumonia, and lung cancer chest diseases. Comput. Biol. Med..

[21] Ghazi F., Benkuider A., Zraidi M., Ayoub F., Ibrahimi K. Neighborhood Feature Extraction and Haralick Attributes for Medical Image Analysis: Application to Breast Cancer Mammography Image. Proceedings of the IEEE International Conference on Wireless Networks and Mobile Communications (WINCOM).

[22] Genc A.C., Arıcan E. (2025). Obesity Classification: A Comparative Study of Machine Learning Models Excluding Weight and Height Data. Rev. Assoc. Med. Bras..

[23] Verzellesi L., Botti A., Bertolini M., Trojani V., Carlini G., Nitrosi A., Monelli F., Besutti G., Castellani G., Remondini D. (2023). Machine and Deep Learning Algorithms for COVID-19 Mortality Prediction Using Clinical and Radiomic Features. Electronics.

[24] Wang Z., Zhao X., Li Y., Zhang H., Qin D., Qi X., Chen Y., Zhang X. (2023). Development and Validation of a Multimodal Feature Fusion Prognostic Model for Lumbar Degenerative Disease Based on Machine Learning: A Study Protocol. BMJ Open.

[25] Bardak B., Tan M. (2021). Prediction of Mortality and Length of Stay with Deep Learning. Proceedings of the 2021 29th Signal Processing and Communications Applications Conference (SIU).

[26] Ince F.M., Alkan Bilik O., Ince H. (2024). Evaluating Mortality Predictors in COVID-19 Intensive Care Unit Patients: Insights into Age, Procalcitonin, Neutrophil-to-Lymphocyte Ratio, Platelet-to-Lymphocyte Ratio, and Ferritin Lactate Index. Diagnostics.

[27] Elghalid R.A.M., Aldeeb F.H.A., Alwirshiffani A., Andiasha A., Mohamed A.A.I. (2022). Comparison of Some Machine Learning Algorithms for Predicting Heart Failure. Proceedings of the 2022 International Conference on Engineering & MIS (ICEMIS).

[28] Dou Y., Dou J., Wang H., Lv J. (2023). Comparison of Three Machine Learning Algorithms for Cardiovascular Disease Prediction. Proceedings of the 7th IEEE Information Technology and Mechatronics Engineering Conference (ITOEC).

[29] Kołodziejczak M.M., Sierakowska K., Tkachenko Y., Kowalski P. (2023). Artificial Intelligence in the Intensive Care Unit: Present and Future in the COVID-19 Era. J. Pers. Med..

[30] Jain P., Aggarwal S., Adam S., Imam M. (2024). Parametric Optimization and Comparative Study of Machine Learning and Deep Learning Algorithms for Breast Cancer Diagnosis. Breast Dis..

[31] Zhou S., Hu C., Wei S., Yan X. (2024). Breast Cancer Prediction Based on Multiple Machine Learning Algorithms. Technol. Cancer Res. Treat..

[32] Mansour W.Y., Thomson R.M. (2023). Haralick Texture Feature Analysis for Characterization of Specific Energy and Absorbed Dose Distributions across Cellular to Patient Length Scales. Phys. Med. Biol..

[33] Jeevitha V., Aroquiaraj I.L. (2025). Haralick Feature-Based Texture Analysis from GLCM and SRDM for Breast Cancer Detection in Mammogram Images. Proceedings of the 2025 4th International Conference on Sensors and Related Networks (SENNET)—Special Focus on Digital Healthcare.

[34] Lokhande N.L., Jaware T.H. (2023). Innovative Approach to Lung Nodule Detection Using Random Walker Segmentation and Texture Analysis on CT Images. Proceedings of the 3rd International Conference on Advancement in Electronics & Communication Engineering (AECE).

[35] Mundra S., Vijay S., Mundra A., Gupta P., Goyal M.K., Kaur M., Khaitan S., Rajpoot A.K. (2022). Classification of Imbalanced Medical Data: An Empirical Study of Machine Learning Approaches. J. Intell. Fuzzy Syst..

[36] Nie T., Yuan Z., He Y., Xu H., Guo X., Liu Y. (2024). Prediction of T Stage of Rectal Cancer After Neoadjuvant Therapy by Multi-Parameter Magnetic Resonance Radiomics Based on Machine Learning Algorithms. Technol. Cancer Res. Treat..

[37] Gupta T., Panda S.P. (2022). A Comparison of Machine Learning Algorithms in Predicting Pulmonary Diseases. Proceedings of the 2022 International Conference on Machine Learning, Big Data, Cloud and Parallel Computing (COM-IT-CON).

[38] Gupta I., Bajaj A., Sharma V. (2025). Comparative Analysis of Machine Learning Algorithms for Heart Disease Prediction. Int. J. Hybrid Intell. Syst..

[39] Wahyuni W., Kusrini K., Setyanto A., Utami E. (2024). Feature Extraction Techniques for Patterned Images: A Systematic Literature Review. Proceedings of the IEEE International Conference on Technology Innovation and Its Applications (ICTIIA).

