# Supplementary material for: Mortality Prediction from Patient’s First Day PAAC Radiograph in Internal Medicine Intensive Care Unit Using Artificial Intelligence Methods
Source: Diagnostics (Basel). 2025 Dec 10;15(24):3138. doi: 10.3390/diagnostics15243138 (PMC12732152; doi:10.3390/diagnostics15243138)
Supplement: Supplementary file 1 [file diagnostics-15-03138-s001.zip › TableS1.pdf]

Supplementary Table S1. Example radiomic and texture feature set.

Feature Name	*Value*
Mean Intensity	0.32509
Variance	0.11464
Skewness	0.23008
Kurtosis	-1.70408
Median Intensity	0.18431
Intensity Range	0.92941
Image Entropy	10.37891
GLCM Contrast	162.22543
GLCM Homogeneity	0.61739
GLCM Energy	0.48541
GLCM Correlation	0.98912
GLCM Dissimilarity	3.73729
GLCM ASM	0.23562
Haralick_1_dir_0°	0.23562
Haralick_2_dir_0°	162.22543
Haralick_3_dir_0°	0.98912
Haralick_4_dir_0°	7456.86196
Haralick_5_dir_0°	0.61739
Haralick_6_dir_0°	166.44819
Haralick_7_dir_0°	29665.22241
Haralick_8_dir_0°	5.24893
Haralick_9_dir_0°	7.01602
Haralick_10_dir_0°	0.00132
Haralick_11_dir_0°	2.85085
Haralick_12_dir_0°	-0.50183
Haralick_13_dir_0°	0.95944
Haralick_1_dir_45°	0.23497
Haralick_2_dir_45°	172.26879
Haralick_3_dir_45°	0.98944
Haralick_4_dir_45°	7448.31453
Haralick_5_dir_45°	0.60882

The figure presents a comprehensive set of radiomic and texture-based features automatically extracted from a posteroanterior chest radiograph. These features include: Histogram-based statistics, such as Mean Intensity, Variance, Skewness, Kurtosis, and Entropy,

 https://doi.org/10.3390/diagnostics15243138

which represent the overall pixel intensity distribution and brightness variability within the lung region.
